# Zolpidem Reduces Hippocampal Neuronal Activity in Freely Behaving Mice: A Large Scale Calcium Imaging Study with Miniaturized Fluorescence Microscope

**DOI:** 10.1371/journal.pone.0112068

**Published:** 2014-11-05

**Authors:** Tamara Berdyyeva, Stephani Otte, Leah Aluisio, Yaniv Ziv, Laurie D. Burns, Christine Dugovic, Sujin Yun, Kunal K. Ghosh, Mark J. Schnitzer, Timothy Lovenberg, Pascal Bonaventure

**Affiliations:** 1 Janssen Research & Development, LLC, San Diego, California, United States of America; 2 Inscopix, Palo Alto, California, United States of America; Centre national de la recherche scientifique, University of Bordeaux, France

## Abstract

Therapeutic drugs for cognitive and psychiatric disorders are often characterized by their molecular mechanism of action. Here we demonstrate a new approach to elucidate drug action on large-scale neuronal activity by tracking somatic calcium dynamics in hundreds of CA1 hippocampal neurons of pharmacologically manipulated behaving mice. We used an adeno-associated viral vector to express the calcium sensor GCaMP3 in CA1 pyramidal cells under control of the CaMKII promoter and a miniaturized microscope to observe cellular dynamics. We visualized these dynamics with and without a systemic administration of Zolpidem, a GABAA agonist that is the most commonly prescribed drug for the treatment of insomnia in the United States. Despite growing concerns about the potential adverse effects of Zolpidem on memory and cognition, it remained unclear whether Zolpidem alters neuronal activity in the hippocampus, a brain area critical for cognition and memory. Zolpidem, when delivered at a dose known to induce and prolong sleep, strongly suppressed CA1 calcium signaling. The rate of calcium transients after Zolpidem administration was significantly lower compared to vehicle treatment. To factor out the contribution of changes in locomotor or physiological conditions following Zolpidem treatment, we compared the cellular activity across comparable epochs matched by locomotor and physiological assessments. This analysis revealed significantly depressive effects of Zolpidem regardless of the animal’s state. Individual hippocampal CA1 pyramidal cells differed in their responses to Zolpidem with the majority (∼65%) significantly decreasing the rate of calcium transients, and a small subset (3%) showing an unexpected and significant increase. By linking molecular mechanisms with the dynamics of neural circuitry and behavioral states, this approach has the potential to contribute substantially to the development of new therapeutics for the treatment of CNS disorders.

## Introduction

Understanding how drugs affect complex neuronal networks is critical for the future of neuroscience drug discovery. Therefore, it is essential to invest in the development and applications of new technologies that will enable researchers to study functional neuronal networks. Recently, the BRAIN initiative outlined a set of experimental techniques that hold the most promise to advance our understanding of brain function and brain disorders [1]. One of the highlighted techniques is calcium imaging of neuronal activity, particularly in behaving animals [1], [2], [3].

Imaging neuronal calcium dynamics in behaving animals with miniaturized integrated fluorescent microscopes takes advantage of several recent break-throughs in technology: using viral vectors to express fluorescent indicators in a targeted genetically identified neuronal population [4]; the use of micro-optics to visualize deep brain structures; and utilization of semiconductor optoelectronics for rapid image acquisition [5], [6]. Using a miniaturized (<2g) integrated fluorescent microscope (nVista, Palo Alto, CA) allows for high-speed imaging at the cellular level of hundreds of neurons in multiple brain regions, including evolutionally conserved deep structures, in freely behaving rodents [5], [6], [7].

This technology, especially if used in combination with other recording techniques, is a transformative new platform for neuroscience drug discovery research. This powerful combination has the ability to efficiently identify compounds that either disrupt normal neuronal activity, or restore normal network activity that was affected by disease, stress or pharmacological manipulations. The traditional drug discovery process is based on a drug’s ability to affect isolated biological targets in artificial systems with subsequent validation in functional and behavioral assays. The latter is often uninformative because the same underlying changes in neuronal networks can have distinct species-specific behavioral effects; conversely, apparently similar behaviors can have different underlying causes. Despite the need to directly investigate drug effects on neuronal activity in freely behaving animals, this step was often omitted because, until recently, techniques lacked the necessary neuronal yield and were not easily integrated into industrial settings. High throughput in-vivo calcium imaging overcomes these limitations.

To demonstrate the potential of this approach for drug discovery, we conducted a proof-of-concept study investigating the effects of Zolpidem on hippocampal neuronal activity measured with the genetically-encoded calcium indicator GCaMP3. Zolpidem was selected for these studies as it (a) is widely used as a therapeutic agent; (b) is pharmacologically well-characterized; (c) has a straightforward behavioral and physiological readout (sleep); (d) has not been characterized in terms of impact on the neuronal activity in behaving animals.

Zolpidem is a short-acting nonbenzodiazepine hypnotic that potentiates GABA transmission by acting on GABA A receptors (reviewed in [8]–[10]). Because GABA A receptors are widespread in the brain, Zolpidem was proposed for use as a therapeutic agent in a wide variety of CNS disorders such as epilepsy [11], [12], anxiety [13]–[17], pain management [18]–[20], deep coma and disorders of consciousness [21]–[27] and many more (reviewed in [28]). It is also one of the most commonly prescribed medications for the treatment of insomnia in the world: in the US alone it accounts for more than 30 million of yearly prescriptions [8]. Growing concern about the adverse effects of Zolpidem has led to reduction in prescribed dosage in some patients and several FDA-issued warnings regarding ataxia, impaired performance, slower reaction time, higher risk for motor accidents, complex abnormal behaviors and amnesia [8], [29]–[31]. The mechanisms of Zolpidem amnesia - specifically, inability to recall episodes that happened while patients were under the influence of Zolpidem - are currently poorly understood [32]. Furthermore, surprisingly little is known about Zolpidem’s effects on neuronal activity in the hippocampus, a structure that is crucial for the normal cognition and episodic memory. While a number of studies investigated effects of Zolpidem on neuronal activity in hippocampal slices [33]–[40], to the best of our knowledge, there are no prior studies investigating Zolpidem effects in hippocampal neurons *in vivo*, let alone in freely behaving animals.

In this study, we used a miniature integrated fluorescence microscope (nVista) to track somatic calcium dynamics of hundreds of hippocampal neurons expressing the calcium indicator GCaMP3 under CamKII promoter. We selected a dose of Zolpidem (10 mg/kg) that closely reproduces sleep-inducing effects in humans: decreased wake time and latency to sleep with specific increases in non-rapid eye movement sleep (NREM) (reviewed in [8], [41]–[42]). After verifying that the imaging procedure did not disrupt the sleep-inducing and sleep-promoting effects of Zolpidem, we investigated effects of Zolpidem on calcium dynamics of individual of CA1 principal hippocampal neurons in freely behaving mice. We found that Zolpidem significantly lowered the frequency of calcium transients in the hippocampus of freely behaving mice. To investigate whether decreases in calcium signaling following Zolpidem treatment could be explained by a decrease in motor activity, we compared the frequencies of calcium transients between the vehicle and drug conditions in the epochs matched by the locomotive states. To further reduce ambiguity in the interpretation of the observed drug effects, we accounted for Zolpidem changes in the vigilance, cognitive and sensory processing factors by studying neuronal activity during physiological (vehicle) and drug-induced (Zolpidem) NREM. We accomplished this step by simultaneous measuring of body temperature, electroencephalogram (EEG), electromyogram (EMG) and locomotor activity concurrently with imaging.

## Methods

### Viral Vector

The University of Pennsylvania Penn Vector Core custom produced AAV2/5 vector expressing GCaMP3 (HHMI/Janelia Farm) via the CaMKII promoter (AAV5.CaMKII.GCaMP3.3WPRE.hGH, titer 9.3e12 GC/ml). After imaging experiments, we verified the levels of GCaMP3 expression in CA1 pyramidal neurons (an example is shown in Fig. S1). In several separate experiments, we used anti-PV (Alpha; clone 2E11; Vector Laboratories Catalog # VP-P963), anti-GABA (Sigma Catalog # A2052; and Sigma Catalog #A0310), GAD67 (Abcam Catalog # ab26116) and anti-CAMK2 (Sigma Catalog # SAB4503244) antibodies to verify GCaMP3 and CaMKII co-expression as well as the absence of expression in the cells expressing some of the common markers of inhibitory interneurons.

### Drug preparation

Before each imaging session Zolpidem (Zolpidem Tartrate BP, Mfg. by M/s. Aarti Drugs Ltd., Tarapur) was dissolved in ultrapure water to 1 mg/ml. The mice were weighed before each imaging session and given an oral gavage of the same volume of vehicle (ultrapure water) and Zolpidem (10 mg/kg).

### Animals

All animal experimental procedures were performed in accordance with the Guide for the Care and Use of Laboratory Animals adopted by the US National Institutes of Health (Janssen IACUC protocol 100–311); the IACUC committee specifically approved this study. Male C56BL/6 mice, aged 8–12 weeks at start, were housed individually with enrichment under controlled conditions with lights on at 6 am, 12∶12 light/dark schedule. The mice underwent two separate surgical procedures under Isoflurane (1.5–2.5%) with analgesic treatment (Buprenex, 0.05 mg/kg sc). During the first surgery, the viral vector was injected (900 nL) into CA1 (AP: −1.9 mm; ML: 1.4; DV: 1.64 mm from Bregma) (Franklin and Paxinos, 2004). Upon full recovery (1–2 weeks later), an optical guide tube was implanted over CA1 [5], [7]. To prevent mechanical compression of the CA1, a cylindrical column of neocortical tissue (∼1 mm^3^) directly above CA1was removed, without disturbing the structure being imaged. During the second surgical procedure, a subset of animals also received a subcutaneously implanted telemetric device (PhysioTel F20-EET; Data Sciences International, St. Paul, MN) for polysomnographic recordings [43]–[44]. In the animals equipped for telemetry, we coupled the devices to two sets of stainless steel electrodes: one implanted in the frontal and parietal cortex for the electroencephalogram (EEG); the second in dorsal nuchal muscles for the electromyogram (EMG). We assessed the quality of the viral expression, the placement of the optical cannula (Fig. S1), electrodes and overall health of the tissue by histological examination after experiments were completed. The animals with incorrect placements, levels of fluorescence or any signs of phototoxic damage (reduced density of cell bodies in CA1, darkening of the fluorescent tissue under the optical cannula, less fluorescence in the cell bodies under the cannula than in the surrounding CA1 tissue etc.) were excluded from the analysis.

The mice were allowed to fully recover (4–6 weeks) before the final preparation for the imaging experiments. First, under Isoflurane (1–1.5%) sedation, the lens (GRINtech GmbH, 0.44 pitch length, 0.47 NA) was affixed into the optical guide tube [5], [7] using UV-curing adhesive (Norland, NOA 81). The microscope (nVista; Inscopix, Palo Alto, CA) was used to assess a suitable imaging site by observing the overall patterns of fluorescence, blood vessels and other landmarks of the site suitable for imaging. Once the GCaMP3 fluorescence in the imaging field was verified under the camera’s LED source, the microscope’s baseplate was permanently fixed to the skull using LED curing cement [7]. The microscope’s baseplate affixed to the skull allowed for repeated attachment of the microscope and reproducible imaging from the same area of the CA1 region of the hippocampus. The microscope was removed between imaging sessions and attached under a light sedation before each imaging session.

Prior to the actual imaging experiment, the mice were habituated for 3–6 days. The habituation sessions were identical to the actual imaging sessions except that the vehicle was dosed and a “dummy” scope (Inscopix, Palo Alto) that mimics the actual microscope’s shape and weight was used.

### Imaging session in open field arena

**Figure 1:**
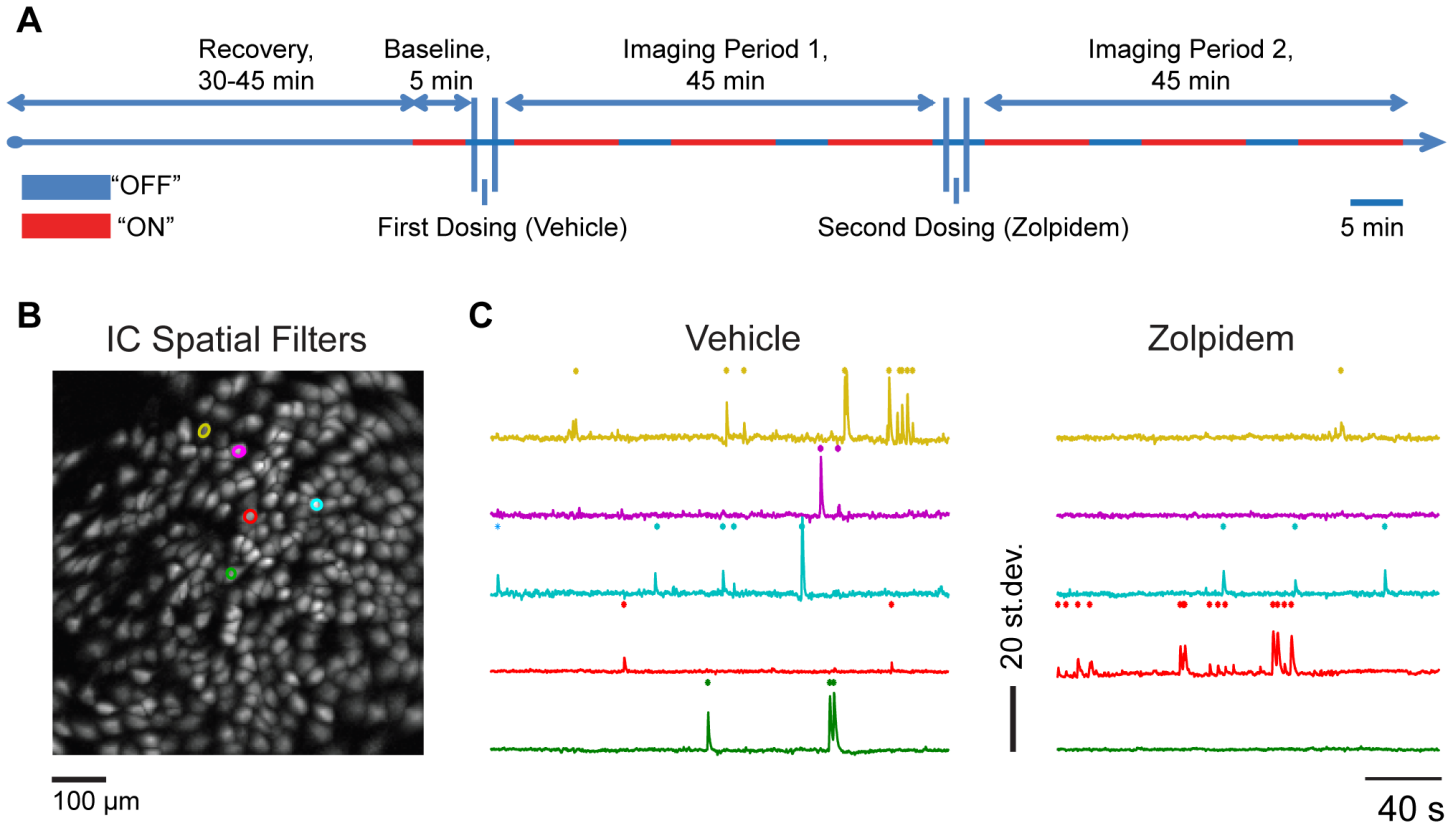
Experimental session and imaging data analysis procedure. **A:** timeline of imaging sessions. We imaged in “interrupted regime” (“On”, red; “Off”, blue) for 45 minutes in vehicle and Zolpidem periods to collect 30 min of neuronal data in each period. **B**: Spatial locations of independent components (ICs) corresponding to the individual cells (“IC Spatial Filters”) identified by PCA/ICA cell sorting algorithm. **C**: Relative fluorescent changes (Δ*F*′(*t*)/*F*′0* = *(*F*′(*t*) – *F*′0)/*F*′0, where *F*′0 is the projected mean intensity for all frames) for five representative cells (ICs, highlighted on panel B and illustrated by Video S1) are calculated and plotted across time. Ca^2+^ transients were identified by searching each trace for local maxima that had peak amplitude more than two standard deviations (st. dev., y-axis) from the baseline (defined as the median of the trace calculated across the entire session); an occurrence of a calcium transient is indicated as a tick mark.

The timing of the imaging session is shown on Fig. 1A. At the beginning of the session, the microscope was attached to the baseplate under brief light anesthesia (<1 min, 0.5–1% Isoflurane). After recovery from sedation (30–45 minutes), the mice were placed in the open field arena and 5 minutes of baseline imaging data were collected. The animals were then dosed with the vehicle, and imaged during 45 minute period. To avoid potential photobleaching, an “interrupted” imaging regime was used whereby the imaging and, correspondingly, LED illumination lasted no more than 10 minutes, with at least 5 minutes between each imaging interval (Fig. 1A). After collecting 30 minutes of post-vehicle data, the animals were dosed with Zolpidem (10 mg/kg s.c.) and imaged over 45 minute period to collect 30 minutes of post-Zolpidem data.

### Imaging session with multimodal recordings

The multimodal recording sessions were the same as in the open field with the following modifications: (1) the imaging was performed while animals were freely behaving in the animal’s home cage placed in a sleep recording chamber [43]–[45]; (2) the post-vehicle and post-Zolpidem 3-hour long sessions were conducted separately (with<one week between sessions) conducted with the “interrupted” regime with each imaging fragment lasting 4 minutes with 2 minute non-imaging intervals between the fragments; and (3) the 10 min baseline (pre-treatment) data was collected while the mice explored a novel circular arena with enrichment and treats. The latter was done to elicit maximal cellular response and facilitate subsequent cellular extraction, identification and alignment across the imaging sessions.

### Sleep recording and analysis

To determine states of vigilance, body temperature, EEG and EMG signals were recorded for 3 hour following vehicle and drug administration and the signals were then digitized (100 Hz sampling rate) using Dataquest A.R.T. software (Data Science International). EEG/EMG recordings were binned into 10 sec intervals and classified according to the vigilance state as previously described [43]–[45]. To verify that the imaging procedure did not disrupt normal and Zolpidem-induced sleep patterns, a separate group of animals not equipped for the imaging was used for the sleep pattern comparison (“Telemetry only” vs “Telemetry+Imaging” animals, Table 1). Analysis of sleep-wake parameters included (a) latency to NREM sleep (defined as the time interval to the first six consecutive NREM epochs during 3 hour post-dosing measurement period); (b) latency to REM sleep (the first two consecutive REM epochs during 3 hour post-dosing measurement period), (c) duration of NREM and REM sleep (minutes per 2 hours of post-dosing). The total wake time was calculated by subtracting the duration of REM and NREM from the total time of the sleep duration measurement period (120 min). To assess the potential impact of combined imaging and telemetry on an animal’s locomotor activity, the ambulatory displacements were detected on the “Activity” channel and were subsequently quantified in 10-sec intervals (“Activity counts”, Table 1; 2 animals were excluded from this analysis due to the questionable quality of the signals on the “Activity” channel). A two-way mixed-design ANOVA (implemented under “anovan” function in Matlab) was used to establish the statistical significance of the factors of drug conditions (Zolpidem vs. vehicle) and protocols (“Telemetry only” vs “Telemetry+Imaging”); and a paired t-test (ttest(X) function in Matlab) was used to compare each parameter within a group.

**Table 1:** Similar sleep-promoting effects of Zolpidem in “Telemetry+Imaging” and “Telemetry only” mice.

		Telemetry only	Telemetry+Imaging
NREM latency	Vehicle	23.4±1.5	28.9±2.1
	Zolpidem**	2.1±0.1	1.8±1.8
REM latency	Vehicle	72.9±5.5	100.4±11.4
	Zolpidem	99.9±8.7	149.8±4.4
Wake duration	Vehicle	46.2±2.9	57.7±13.8
	Zolpidem**	28.7±5.5	32.6±7.0
NREM duration	Vehicle	68.8±0.9	59.9±2.2
	Zolpidem**	88.5±1.0	87.4±1.4
REM duration	Vehicle	5.0±0.7	2.5±0.8
	Zolpidem	2.8±0.6	0±0
Activity counts	Vehicle	878±136	768±104.6
	Zolpidem**	368±11	230±48

Duration of Wake, NREM and REM sleep, and Activity counts were measured during the first 2 hour period after oral dosing of Zolpidem (10 mg/kg). Latency to NREM and REM sleep were measured during the first 3 hour period. Measurements are expressed in minutes and represented as means ± s.e.m. (n = 5 animals per condition).

*p<0.05 and **p<0.01; comparison assessed by two-way mixed-design ANOVA.

For the analysis of neuronal activity during NREM, the epochs were selected according to the following criteria: a 4 min bin of uninterrupted unambiguous NREM which consisted of 24 uninterrupted 10-sec bins classified as “NREM” and was flanked by at least 4 of consecutive 10-sec NREM bins. The alignment between the imaging system and multimodal data collection was done by recording, on a separate channel, the state of the imaging system transmitted through an analog channel in nVista and subsequently digitized at 100 Hz.

### Behavioral recording and analysis

An overhead camera connected to an automated video tracking system (VideoTrack; ViewPoint Behavior Technology) on a PC computer recorded and digitized (30 Hz sampling rate) the x- and y-position in a 50 × 50 cm arena. The system automatically detected the large ambulatory movements (>7.2 cm/min), small movements (<7.2 cm and >0.2 cm/min) and inactivity (<0.2 cm/min). The velocity (measured as displacements, in cm, over periods of time, in minutes) was calculated in 1-sec bins. Alignment between the imaging and the video tracking systems was done by triggering imaging through the VideoTrack software.

### Processing and analysis of calcium imaging videos

A custom in-house Matlab script was used to preform image analysis as we briefly describe here. Since the original high-definition data provided finer spatial resolution than necessary for analysis of cellular activity, a spatial down-sampling of 16x was applied and the image cropped to visualize the area containing optimal cellular signal activity. Correction for the slight lateral displacement (motion artifact) was achieved by applying an image registration (ImageJ plugin TurboReg implemented under the MatLab interface) to align all movie frames to a single movie target. To identify the individual cells, the registered images were expressed as relative change in fluorescence, Δ*F*′(*t*)/*F*′0* = *(*F*′(*t*) – *F*′0)/*F*′0, where *F*′0 is the projected mean intensity for all frames. Spatial filters corresponding to individual cells were identified using a cell-sorting algorithm that applies principal and independent component analyses [5]–[7], [46]. Cells’ spatial filters were based on Ca^2+^ activity over the entire session. Each cell’s thresholded spatial filter was used to extract the individual cell’s Ca^2+^ activity from the Δ*F*′(*t*)/*F*′0 stack. The Ca^2+^ transients were identified by searching each trace for local maxima that had peak amplitude more than two s.d. from the baseline (defined as the median of the trace calculated across the entire session) for at least 0.5 sec at a separation of 300 ms from adjacent Ca^2+^ transients.

### Statistical analysis of neuronal data

The calcium transients event rate for each individual cell was calculated across both treatment conditions (vehicle and Zolpidem) to determine the effect of Zolpidem on individual cells and population activity. To compare event rates across drug conditions (vehicle vs Zolpidem), the Wilcoxon Signed Rank Test (WSR) was used on the data pooled across cells and binned into 1-minute intervals. To take into account the number of animals used in the experiments, and the variations between animals, we also performed a one-tailed WSR tests on the averaged values obtained for each animal thus using sample “n” as number of animals rather than the number of cells. To compare neuronal activity during active and inactive periods, 1-minute intervals containing at least one large ambulatory movement (>7.2 cm/min displacements) were designated as active, while 1-minute intervals during which total displacements were less than 0.2 cm were designated as inactive. To study Zolpidem effects on the level of individual cells, the event rates (calculated in 2-minute bins) were compared using Mann-Whitney-Wilcoxon (MWW) test. Initially, cells with a significant change (p<0.05) were identified; these cells were then subdivided into two groups depending on the direction of change (increased or decreased event rate). To address the concern that the observed drug effects on the level of individual cells are not above the level expected by chance, we first calculated the normalized drug index for each cell: (post-drug event rate - post-vehicle event rate)/(post-drug event rate+post-vehicle event rate). The expected (if Zolpidem had no effect) distribution of drug indices was then constructed by re-sampling (with replacement) post-vehicle bins within each cell (1000 shuffles, “bootstrp” Matlab function). A cell was considered to have a significant effect if its index value was outside the 99% confidence interval for the expected distribution.

## Results

### Imaging procedure did not disrupt the sleep-promoting effects of Zolpidem

To assess whether the imaging surgical preparations and imaging procedures (anesthesia, microscope attachment, illumination, tethering etc.) disrupted the sleep-promoting effects of Zolpidem, we conducted a study to compare the effects of Zolpidem in a group of animals in which we conducted telemetry (EEG/EMG and locomotor activity) recordings only (“Telemetry only”) to another group in which telemetry was conducted at the same time as calcium imaging (“Telemetry+Imaging”). When orally dosed at the beginning of the light phase, Zolpidem (10 mg/kg) was effective in inducing and prolonging sleep duration in both groups of mice. As shown in Table 1, Zolpidem similarly shortened the latency for NREM sleep in “Telemetry only” (−91%) and “Telemetry+Imaging” (−94%) groups as compared to vehicle treatment (two-way mixed-design ANOVA; significant drug effect: Zolpidem vs vehicle, p<10^−4^; non-significant effect of the protocol: “Telemetry only” vs. “Telemetry+Imaging”, p = 0.39; non-significant interaction, p = 0.34). In a comparable manner, the time spent in wake was reduced in “Telemetry only” (−38%) and “Telemetry+Imaging” (−44%) groups (two-way mixed ANOVA; significant drug effect: p<10^−4^; non-significant effect of the protocol: p = 0.09; non-significant interaction, p = 0.33); whereas NREM sleep duration was increased in “Telemetry only” (+29%) and “Telemetry+Imaging” (+46%) groups during the first 2 hours after dosing relative to vehicle treatment (two-way mixed ANOVA; significant drug effect: p<10^−4^; non-significant effect of the protocol: p = 0.14; non-significant interaction, p = 0.25). Zolpidem also reduced locomotor activity in both groups (two-way mixed ANOVA performed on activity counts; significant drug effect: p = 0.0096; non-significant effect of the protocol: p = 0.4462; non-significant interaction, p = 0.93). Changes in the REM sleep state were not significant following zolpidem dosing vs. vehicle in both groups, although a tendency toward an increase in REM latency and a decrease in REM duration were observed.

### Zolpidem decreased the frequency of calcium transients in CA1

**Figure 2:**
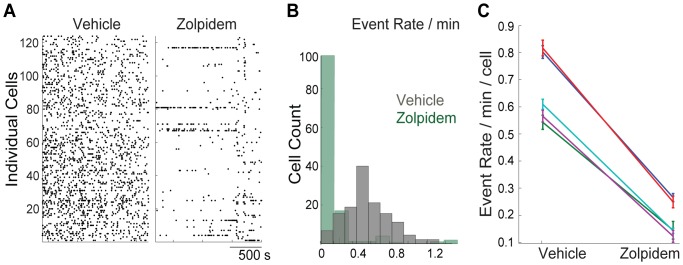
Zolpidem decreased frequencies of calcium transients in CA1. **A**: Calcium transients (indicated by tick marks) detected in individual cells (vertical axis) are plotted across time following vehicle (water, left) and Zolpidem (10 mg/kg, right) administration in a representative animal. **B**: Histogram of calcium transients (“Event Rate”) in the representative animal. **C**: Average rate of calcium transients (s.e.m. error bar) in all animals used in the study. Zolpidem decreased the frequency of calcium transients by 71% (from 0.7120 to 0.2087 events/min/cell, p<0.0001, Wilcoxon Signed Rank Test).

The timing of a typical experimental session used to collect imaging videos of the fluorescent cellular signal after oral administration of the vehicle (water) and Zolpidem (10 mg/kg) is shown on Fig. 1A. The Videos S1 and S2 show example of an imaging session following treatment with vehicle and Zolpidem. From the recorded imaging videos, the location of each individual cell was extracted (Methods, “Processing and analysis of calcium imaging videos”; Fig. 1B) and the individual calcium dynamics assessed across the session (Fig. 1C). The distributions of detected individual calcium transients (Methods, “Processing and analysis of calcium imaging videos”) were statistically compared between vehicle and drug conditions (Methods, “Processing and analysis of calcium imaging videos”; an occurrence of a calcium transient is indicated as a tick mark on a raster plots on Fig. 1C, “Events” and Fig. 2A). The frequency of calcium transients detected following Zolpidem administration was lower in comparison to vehicle (Fig. 2A: a raster plot of a representative session; Fig. 2B: distribution of calcium transients in vehicle and Zolpidem periods in the example session; Fig. 2C: summary plot of all sessions). In the data combined across 5 recording sessions, Zolpidem decreased the frequency of calcium transients by 71% (from 0.7120 to 0.2087 events/min/cell, p<0.0001, Wilcoxon Signed Rank Test).

### Post-Zolpidem decrease in the frequency of calcium transients was not a by-product of post-Zolpidem decrease in motor activity

The animals’ locomotor activity was continuously monitored during the imaging sessions. Quantification of the animals’ displacements over the course of the session was carried out in 1 second time bins. In confirmation of previous studies, Zolpidem significantly lowered the animals’ locomotor activity (MWW test on distribution of instances of displacements exceeding 0.2 cm/min counted in 1-min bins; p<0.0001, n = 5 animals). Correspondingly, there was a significant difference in frequencies of calcium transients between active and inactive periods in vehicle (active: 0.8172±.0163; inactive: 0.6287±0.0206; WSR test, p<.001) and Zolpidem conditions (active: 0.3695±.0214; inactive: 0.1981±.0146; mean average value given in units of events/min/cell for each condition ± s.e.m; correspondence between average frequency of calcium transients and average speed is illustrated in Fig. S2).

**Figure 3:**
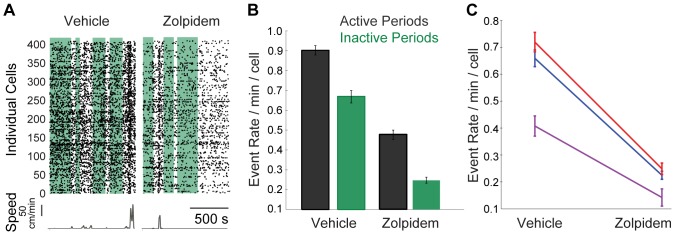
Decrease in locomotion was not sufficient to explain Zolpidem-induced decrease in neuronal activity. **A**: Raster plot of calcium transients in individual cells (vertical axis) following vehicle (left) and Zolpidem (10 mg/kg, right) administration in an example animal with identified inactive (displacements <−.2 cm/min) periods (green shading). The corresponding speed (in cm/min) trace is plotted below the raster plot. **B**: Comparison of average frequencies of calcium transients (number of events/minute/cell) during active periods (black bars) and inactive periods (green bars) in the representative animal (s.e.m. error bar). **C**: Average rate of calcium transients (s.e.m. error bar) in all animals with identified inactive periods following both vehicle (left) and Zolpidem (right) administration.

To account for the contribution of decreased locomotion to the observed neuronal effects, we compared the frequency of calcium transients (vehicle vs. Zolpidem) measured in 1-minute bins during which the animals were inactive; inactivity bins were defined as bins with total displacements of less than 0.2 cm. An example of a typical imaging session demonstrating this approach is shown on Fig. 3A. The animal’s speed is plotted as a function of time below the corresponding raster plot of calcium transient events; the identified inactive periods are indicated as green shading overlaid on the raster plot. In this session, the rate of detected calcium transients was counted only during inactive Zolpidem periods and was significantly lower than during inactive vehicle periods (WSR test, p<0.0001; Fig. 3B). The suppressive effect of Zolpidem on the frequency of calcium transients was consistent across sessions within the identified inactive periods, and this effect was highly significant in the data combined across sessions (WSR test, p<0.0001, n = 943 cells, Fig. 3C). On average, rate of detected calcium transients dropped from 0.60 events/min/cell in post-vehicle inactive periods to 0.20 events/min/cell in post-Zolpidem inactive periods (67% decrease). This result indicated that Zolpidem decreased net neuronal activity in the hippocampus beyond what is expected from decreased locomotion.

### Individual hippocampal CA1 pyramidal cells differed in their responses to Zolpidem

**Figure 4:**
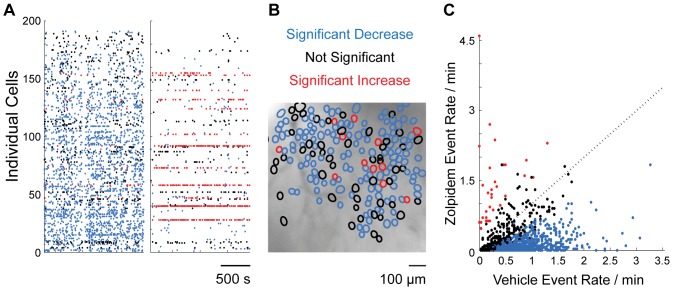
Individual hippocampal CA1 pyramidal cells differed in their responses to Zolpidem. **A**: Representative raster plot of calcium transients in individual cells (n = 195) that are color-coded depending on their response to Zolpidem (Mann-Whitney-Wilcoxon test, p<0.05 criterion of significance): significant decrease (blue); significant increase (red), non-significant change (black). **B:** Locations of individual cells identified in the same representative imaging session, layered atop a mean fluorescent image. **C**: Rate of calcium transients post-Zolpidem (“Zolpidem Event Rate”) vs post-vehicle (“Vehicle Event Rate”); each dot is an individual cell (n = 1275). The majority of individual neurons (65%) significantly lowered neuronal activity following Zolpidem administration; 32% of neurons did not show a significant change; and a small neuronal subset (∼3%) showed a significant increase.

We assessed the changes in rate of calcium transients between vehicle and Zolpidem imaging periods for each individual cell (Methods, “Statistical analysis of neuronal data”). Cells that showed a significant Zolpidem effect (MWW test, p<0.05) were subdivided into two groups depending on the direction of change (increased or decreased event rate). The approach is illustrated on the raster plot on Fig. 4A: in this representative session, the majority of individual cells (shown in blue) decreased their activity following Zolpidem administration relative to the vehicle, while some of the cells either increased their activity (shown in red) or had no significant change (shown in black, p>0.05, MWW test). Fig. 4B illustrates spatial positions for the cells within each class.

This result was consistent across the sessions: the majority of individual neurons significantly lowered the frequency of calcium transients following Zolpidem administration (as exemplified by a majority of points falling below the unity line on the scatter plot shown on Fig. 4C): 65% (829 out of 1275) of individual neurons significantly decreased activity, while 32% of neurons (405 out of 1275) did not show a significant change. A small neuronal subset (∼3%, 41 out of 1275; shown in red on Fig. 4 A–C) significantly increased their activity following Zolpidem administration. To assess whether the effect of significant increase was a result of a type I statistical error (false positives), we performed the following additional analysis. We first calculated a normalized drug index for each cell: (post-drug event rate - post-vehicle event rate)/(post-drug event rate+post-vehicle event rate). The expected (if the drug had no effect) distribution of drug indices was constructed by re-sampling (with replacement) vehicle bins within each cell (1000 shuffles, “bootstrp” Matlab function) (Fig. S3A). The distribution of the constructed indices was centered on zero, as was expected if there was no difference in calcium transients’ rate between treatment conditions. The observed distribution of actual indices (Fig. S3B) was heavily shifted toward extreme negative values of the index (“−1”), indicative of much lower activity following Zolpidem administration; however, the indices formed a continuous range with a monotonic rightward decrease (toward “+1” values of the index) with some of the cells in our sample (18 out of 1275) having index values exceeding 99% confidence interval of the expected distribution. This result confirmed that individual pyramidal neurons in CA1 differed with respect to their response to Zolpidem, forming a continuous range of the responses with the predominant effect of decreased activity following drug treatment.

### Zolpidem suppressed the frequency of calcium transients during NREM sleep

**Figure 5:**
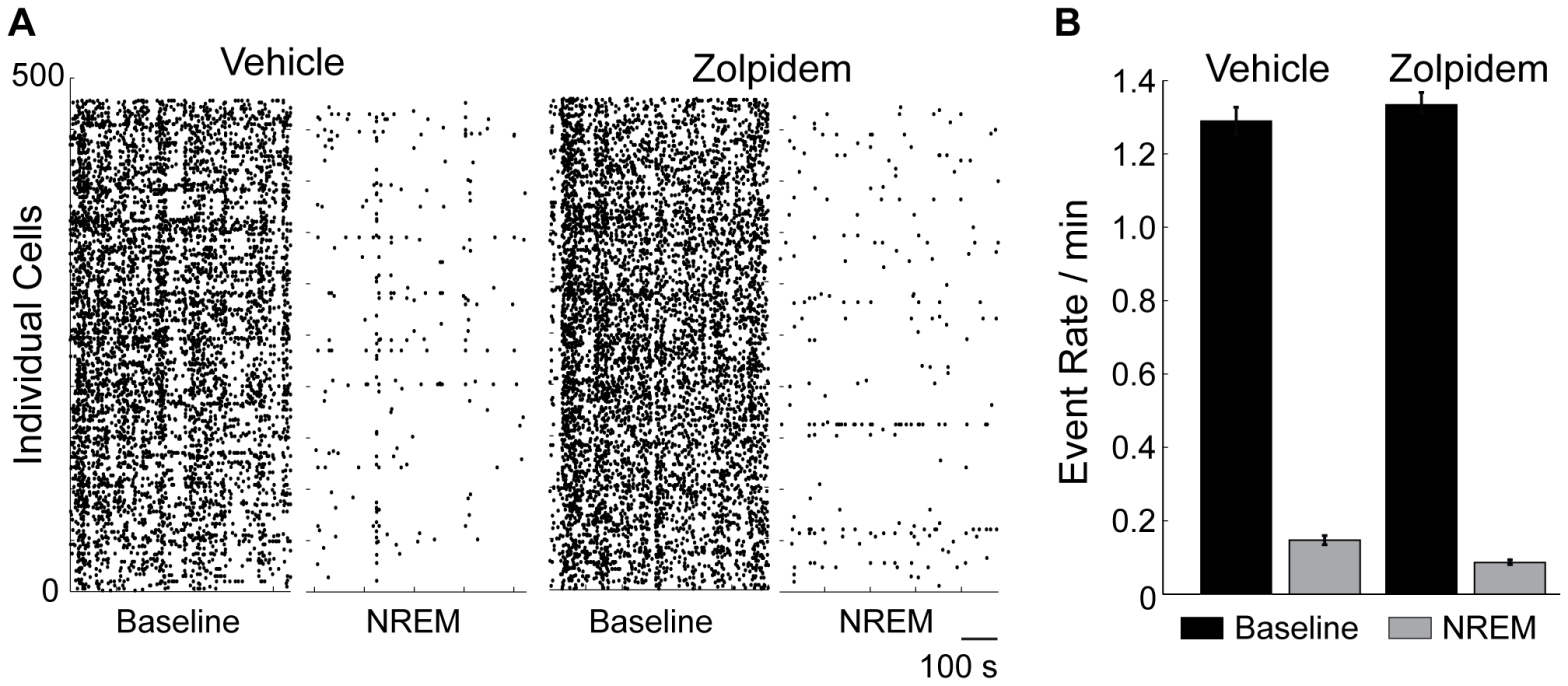
Neuronal activity during Zolpidem-induced NREM sleep was lower than neuronal activity during physiological NREM. **A**: Raster plots of calcium transients in 478 individual cells (vertical axis) during pre-treatment active wake periods (“Baseline”) and post-treatment NREM periods (“NREM”) in two imaging sessions (“Vehicle” and “Zolpidem”). **B:** Average frequencies of calcium transients (“Event Rate”: number of events/minute/cell) during pre-treatment active wake (in both Vehicle and Zolpidem sessions, black bars), physiological NREM (“Vehicle”, grey bar) and Zolpidem-induced NREM (“Zolpidem”, grey bar). The error bars are the s.e.m. for each condition across all cells. Zolpidem NREM neuronal activity was significantly lower than vehicle NREM neuronal activity (0.09 and 0.15 events/minute/cell, respectively, 40% change, p<0.004, WSR test).

To factor out contribution of differing physiological states on the observed drug effects, we compared the frequency of calcium transients between vehicle and Zolpidem imaging periods in the epochs matched by an identified physiological state (NREM), the duration of which was specifically increased by Zolpidem (Table 1). Prior experiments indicated that 3 hour imaging session would be necessary for the appropriate statistical comparison of neuronal data during continuous unambiguous NREM between Zolpidem and vehicle condition (when animals spent less time in NREM). To minimize potential photobleaching, we conducted the experiment in 2 separate 3-hour long sessions (Methods, “Imaging with multimodal recordings”). We tracked activity of the same individual cells (n = 478) in both sessions: the vehicle session (Fig. 5A, “Vehicle”) and Zolpidem session (Fig. 5A, “Zolpidem”). To facilitate identification and alignment of individual cells across sessions, we collected 10 minutes of pre-treatment baseline data while animals were actively exploring a novel environment (Fig. 5A, “Baseline”). The frequency of calcium transients during baseline periods was significantly different across sessions (1.33 events/minute/cell in Zolpidem session vs 1.29 in vehicle session; WSR test, p<0.001), most likely due to the variations in the animal’s exploratory activity or other behavioral factors.

The frequency of calcium transients during NREM was substantially lower than during active exploration (baseline) in both vehicle and Zolpidem conditions (WSR test, p<0.001; Fig. 5B; average event rates during baseline and NREM are shown as black and grey bars, respectively). During the vehicle session, activity dropped from 1.29 to 0.15 events/minute/cell (89% decrease; Fig. 5B, left). In the Zolpidem session, the frequency of calcium transients dropped from 1.33 to 0.09 events/minute/cell (93% decrease; Fig. 5B, right). It is important to note that while differences in the frequency of calcium transients during the active wake (baseline periods) of the two different sessions could reflect a difference in behavioral and cognitive factors of active wake, during continuous unambiguous NREM (Methods, “Sleep recording and analysis”) contributions of sensory, cognitive, emotional and locomotive states are minimal. Nevertheless, the frequency of calcium transients during Zolpidem NREM was significantly lower than during vehicle NREM (0.09 and 0.15 events/minute/cell, respectively, 40% change, p<0.004, WSR test; Fig. 5B, gray bars). This finding further reinforces our conclusion that Zolpidem suppressed calcium fluorescent signals in memory-related structures to below normal physiological levels.

## Discussion

Visualizing neuronal activity with high resolution has until now been extremely challenging in freely behaving animals. Here, we used large-scale calcium-imaging with a miniaturized fluorescence microscope to study effects of a pharmacological agent in the hippocampus of freely behaving mice. This technique allows investigation of the effects of pharmacological agents in (a) deep, evolutionary preserved homologous structures such as hippocampus; (b) relevant brain circuits with largely preserved natural connectivity within those circuits; (c) after systemic administration of a drug; (d) while simultaneously observing drug’s effect on animal’s behavior and physiology. While it is possible to achieve (a)-(d) by other techniques such as multi-electrode array recordings, calcium imaging is advantageous for the drug discovery settings because it has higher neuronal yield and allows investigation of drug effects in genetically identified neuronal populations [2]–[5].

We investigated the effects of Zolpidem, a commonly used treatment for insomnia, on the calcium dynamics of neurons expressing GCaMP3 calcium indicator in the hippocampus, a brain structure that is critically involved in memory, cognition and conscious experience (reviewed in [49]–[59]). We selected the dose of Zolpidem (10 mg/kg) that in mice evokes sleep effects similar to the effects of therapeutic doses in humans: decreased wake duration, increased sleep duration with specific increase in NREM, and reduced latencies to sleep with specific reduction in NREM latency (reviewed in [8], [9], [42]). We verified that the imaging procedure did not disrupt sleep-inducing and sleep-promoting effects of Zolpidem (Table 1).

A novel, to the best of our knowledge, result in this study is that Zolpidem (10 mg/kg, p.o.) decreased activity of hippocampal CA1 pyramidal neurons of freely behaving mice (Fig. 2C): the rate of detected calcium transients after systemic administration of Zolpidem dropped 3-fold relative to the vehicle. This result is in line with a recent study that investigated the effects of a local application of Zolpidem on neuronal activity in somatosensory cortex of anesthetized mice [61]: the authors found that evoked neuronal calcium activity decreased approximately 3-fold following Zolpidem application (10 µM). Similarly, Zolpidem (1 mg/kg, i.v.) administration led to a 3–4 fold decrease in firing rate of medial septum neurons of anesthetized rats [62]. The alignment between the effects observed in anesthetized and freely behaving animals addresses the concern that the previously observed Zolpidem effects could be a by-product of the interaction between Zolpidem and anesthetics [60]–[61]. The tendency of Zolpidem to suppress neuronal activity *in vivo* is in agreement with the findings in hippocampal slices where Zolpidem was consistently found to potentiate inhibitory currents [34]–[40], [63]. It is generally assumed that increased inhibitory currents detected in hippocampal slices would translate into decreased activity of hippocampal cells in behaving animals; our study provides direct evidence in support of this hypothesis.

The suppression of calcium fluorescent signals following Zolpidem administration exceeded levels expected from decreases in locomotion (Fig. 3B–C). It was important to take this potential confounding factor into account because (a) it is known that neuronal activity in the hippocampus is related to the animal’s locomotive state [64]–[67]; specifically, the probability of a neuron’s discharge increases as a function of velocity [47]; and (b) Zolpidem is known to lower locomotor activity in rodents [12], [48], [68]–[72] and impair motor performance in humans [73]–[84]. Here, we employed a simple and conceptually straightforward method to factor out the contribution of locomotion by selecting time periods during which freely behaving mice were inactive. This was possible due to this technique’s high neuronal yield which allowed enough statistical power to meaningfully compare neuronal data collected even over the short periods of time. Our finding that Zolpidem significantly lowered the frequency of calcium transients relative to the vehicle during periods matched by locomotion (Fig. 3A–C) indicated that the effect of Zolpidem observed in the hippocampus is not a simple by-product of decreased locomotion caused by pharmacologically induced changes elsewhere in the brain, for example, motor cortex [85]–[86] or basal ganglia [87]. Instead, it is likely that the decreased locomotion can be in part a consequence of Zolpidem’s suppressive action in the hippocampus. This suggestion is in line with previous work [88], which demonstrated that direct intra-hippocampal injection of Zolpidem (10 µg/site) caused marked decrease of locomotion in rats.

It is important to characterize drug effects at the level of individual neurons as studying exclusively net changes could lead to misinterpretations of the pharmacological effects. For example, a given post-drug change in net neuronal activity can be due to some minor yet consistent changes across individual cells, or because of the major changes in a minority of neurons. Furthermore, pharmacological agents can differentially influence activity of distinct neuronal subpopulations. In this case, no changes in net neuronal activity can either mean that a drug doesn’t have an effect or that a drug evokes opposite responses in different neuronal populations. These cases can be easily differentiated by studying calcium dynamics on a level of genetically identified individual cells. To demonstrate this approach, we characterized the effects of Zolpidem at the level of individual pyramidal (excitatory) CA1 hippocampal neurons, taking advantage of a selective promoter CaMKII. While the net effect of Zolpidem on calcium fluorescent signaling was predominantly suppressive, individual hippocampal pyramidal neurons in CA1 differed with respect to their responses to Zolpidem (Fig. 4A–C). We broadly classified individual cells into two categories depending on whether the distributions of calcium transients in post-vehicle and post-Zolpidem periods differed significantly. The majority (68% of cells) showed significant change (MWW test, p<0.05). We then further subdivided significant cells into 2 categories depending on the direction of the change. The majority of neurons with significant change (829 out of 870) decreased their activity following Zolpidem administration; while a small subpopulation of neurons (41 out of 870) significantly increased their activity.

Because the pharmacology of Zolpidem has been extensively characterized [89]–[90], it is possible to explain the mechanism by which Zolpidem decreases the activity of individual CA1 pyramidal neurons. Zolpidem is a positive allosteric agonist of GABAA α1-containing receptors (reviewed in [8]–[10], [91]–[95]) that mediate influx of chloride ions and thus hyperpolarize neurons. GABAA α1-containing receptors mediate the bulk of inhibitory drive in mature mammalian brain (reviewed in [42], [93], [96]–[97]) and are expressed at a high level in most of the neurons in CA1 region of the rodent hippocampus [97]–[104]. Therefore, in the majority of CA1 pyramidal neurons that express GABAA α1-containing receptor, Zolpidem would potentiate inhibitory currents. That, in turn, would result in the decreased activity, as directly observed in our experiments. Correspondingly, the neurons that did not show a significant change post-Zolpidem presumably lack α1 subunit containing GABAA receptors. From the *in vitro* literature [104]–[107], we estimated that ∼30% of hippocampal CA1 pyramidal cells lack α1 subunit containing GABAA receptors; this number closely matches 32% of neurons with no significant Zolpidem effect in our sample.

While Zolpidem predominantly lowered the frequency of calcium transients, some neurons (∼3%) responded with a marked increase in their activity (rasters on Fig. 2 and Fig. 4), which means that in some neurons a GABA agonist shifted the balance toward excitation. Since this result appeared somewhat counterintuitive given pharmacological properties of Zolpidem, we interrogated some of the possible explanations. After investigating each animal separately, we confirmed the presence of this effect in all animals. We also verified the placement of each optical cannula over the CA1 region with an intact cellular layer. The cells with increased activity following Zolpidem were randomly interspersed across the entire field (Fig. 4B), and there was no apparent clustering in the parts of the optical cannula most susceptible to the inflammatory damage (cannula’s periphery) or phototoxic damage (center of the cannula). We also ruled out the possibility that the presence of individual “increasing” cells was the result of a type I statistical error (false positives) by an additional analysis in which we compared the observed distribution of the cellular responses to the distribution expected if Zolpidem had no effect (Results, “Individual hippocampal CA1 pyramidal cells differed in their responses to Zolpidem”; Fig. S3). We suggest that the observed increase in activity-related fluorescent signals, rather than being a direct pharmacological action of Zolpidem on individual CA1 neurons that expressed Zolpidem-sensitive receptors, could be a network phenomenon. For example, it could be a consequence of the changed speed of signal propagation in a recurrent network [108], or otherwise changed network dynamics that would shift activity of some neurons toward a bursting regime that is more detectable by the GCaMP3 sensor [7], [46]. This can be tested experimentally by using a calcium sensor that can reliably detect individual spikes [109]–[110]. The most likely explanation, however, is that post-Zolpidem increased activity is a consequence of a release from tonic inhibition that exists, although seems quite rare, in CA1 pyramidal neurons [101], [111]–[119]. Zolpidem could suppress sensitive interneurons that provide tonic inhibition to neighboring pyramidal neurons, which would result in an increased activity. This idea can be tested in future calcium-imaging studies by computing correlations between pyramidal-interneuron pairs expressing differently colored calcium indicators.

One of the key points of this study was to introduce the multimodal recording approach (simultaneous measurement of body temperature, electroencephalographic, electromyographic and locomotor activity concurrently with calcium imaging) to drug discovery and to demonstrate that this approach allows for in-depth investigation of the connections between changes in the calcium signals and the physiological parameters commonly associated with the drug action. We compared calcium dynamics of individual hippocampal cells following vehicle or Zolpidem administration during NREM sleep. We selected this period because (a) Zolpidem specifically increases NREM, as was observed in current study (Table 1) and previous studies [9], [120]–[123]; and (b) NREM is associated with natural locomotor, behavioral and cognitive inactivity and sensory disconnection from the environment (reviewed in [41]–[42]). It was important to factor out the contribution of locomotor, behavioral, cognitive and sensory changes from changes in post-Zolpidem hippocampal calcium fluorescent signals because (a) hippocampal activity is known to be influenced by all of these factors [47], [49]–[59]; and (b) these factors are known to be changed by Zolpidem (recently reviewed in [28]).

The NREM sleep was associated with a low rate of detected calcium transients in CA1 (Fig. 5A–B). This result was expected, because one of the pronounced and diagnostic features of NREM is the presence of the “down” states linked to reduced neuronal activity in multiple brain regions including the hippocampus (reviewed in [41], [124]–[129]). The novel finding in this experiment was that Zolpidem further suppressed hippocampal calcium signals during NREM. Additional studies are needed to investigate whether the changes in hippocampal neuronal activity during post-Zolpidem NREM are connected to some subtle changes in the EEG spectral characteristics [130]–[132].

This study shows that it is now feasible to investigate the effects of pharmacological agents on activity of large number of genetically identified neurons in relevant brain circuits in freely behaving animals with concurrent measure of behavioral and physiological parameters. By linking molecular mechanisms, neural circuit dynamics and animal behavior, this approach has the potential to deepen our understanding of normal and abnormal brain function and how brain networks are impacted by pharmacological agents. For example, the observed suppression of hippocampal activity following Zolpidem administration provides a direct experimental evidence for a simple mechanistic explanation of adverse effects of Zolpidem on episodic memory [13], [16], [28], [73]–[74], [133]–[140]: Zolpidem, when administered systemically in doses that promote sleep, prevents normal functioning of the hippocampus, a structure critically involved in the episodic memory, by lowering below physiological level activity of the majority of hippocampal principal neurons that express GABA A receptors. In addition to the early screening for the potential cognitive side effects on the level of neuronal networks in behaving animals, this approach may have much wider implications for the neuroscience drug discovery process. Currently, the general direction of the field is to find highly selective compounds that interact with a single molecular target. The current early stage process involves compound screening on the level of isolated molecular targets in artificial systems with subsequent validation in behavioral and functional assays in preclinical species. The major difficulty in the process is a very low efficiency and a weak translation from the early stages to the later stages, most notably from preclinical to clinical studies. This could be due to the following factors: (a) an involvement of a given molecular target in a given physiological or pathophysiological process is often partial or just hypothetical; (b) the effects on the level of overexpressed targets in artificial systems are often distinct from the effects in a living organism of a preclinical animal; (c) the behavioral expression of a given condition, especially in case of psychiatric and mood disorders, could be different between preclinical animals and humans despite analogous underlying neuronal network physiology or pathology; and (d) it is possible that a particular behavioral or physiological expression of a disorder could only be reversed by acting on multiple molecular targets. The latter could explain why some of the very effective compounds against a given disorder lack selectivity and thus influence function of multiple molecular targets. Not surprisingly, such compounds were discovered mostly by serendipity [141] because the current drug discovery process discourages discovery of such agents. Studying drug action at the neuronal network level will favor discovery of compounds that reverse pathological neuronal network states regardless of particular molecular selectivity or species-specific behavioral expressions. The approach used in this study could be used for the early screening of the compounds at the level of relevant neuronal networks. Combined with concurrent monitoring of behavioral and physiological parameters, this novel platform could become a valuable resource for identifying and cataloging network signatures of different brain disorders and their behavioral expressions in preclinical species. We believe that this approach promises to bypass the current limitations of drug screening process and has the potential to contribute to the discovery of new breakthrough treatments for CNS disorders.

## Supporting Information

Figure S1 **Histological examination of the tissue following the imaging procedures. A:** A representative example of placement of the optical cannula over CA1 region of the hippocampus (coronal slice, scale bar: 180 µm). Green: GCaMP3 fluorescence. Blue: DAPI staining. **B**: A representative example of the GCaMP3 fluorescence (green) under the optical cannula (scale bar: 88 µm).
(TIF)

Figure S2 **Average rate of calcium transients in all animals used in the study plotted versus average speed of the animals in each condition (indicated by the shape of the marker; vehicle: circles; Zolpidem: squares).**
(TIF)

Figure S3 **Distributions of normalized drug index: (post-drug event rate - post-vehicle event rate)/(post-drug event rate+post-vehicle event rate). A**: The distribution of drug indices expected if Zolpidem had no effect was constructed by re-sampling, with replacement, vehicle data bins within each cells (1000 shuffles). **B**. The observed distribution of drug indices calculated for each cell (n = 1275). Vertical line indicates 99% confidence interval (3 s.d.) calculated from the expected distribution.
(TIF)

Video S1 **Representative videos of imaging sessions with vehicle and Zolpidem illustrate Ca^2+^ transients identification applied to five representative traces shown on** **Fig. 1C** **.** Ca^2+^ transients were identified by searching each trace for local maxima that had peak amplitude more than two standard deviations (st. dev., y-axis) from the baseline (defined as the median of the trace calculated across the entire session); an occurrence of a calcium transient is indicated as a tick mark.
(ZIP)

Video S2 **Representative movies of imaging sessions with vehicle and Zolpidem (in the absence of ambulatory movements in both conditions).** The two movies (shown side-by-side; Vehicle on the left and Zolpidem on the right) display relative change in fluorescence (Δ*F*′(*t*)/*F*′0; shown on 5% scale and sped-up 10 times) recorded in the same 640×570 µm^2^ imaging field.
(ZIP)
