# Supplementary figures and images for: Zolpidem Reduces Hippocampal Neuronal Activity in Freely Behaving Mice: A Large Scale Calcium Imaging Study with Miniaturized Fluorescence Microscope
Source: PLoS One. 2014 Nov 5;9(11):e112068. doi: 10.1371/journal.pone.0112068 (PMC4221229; doi:10.1371/journal.pone.0112068)

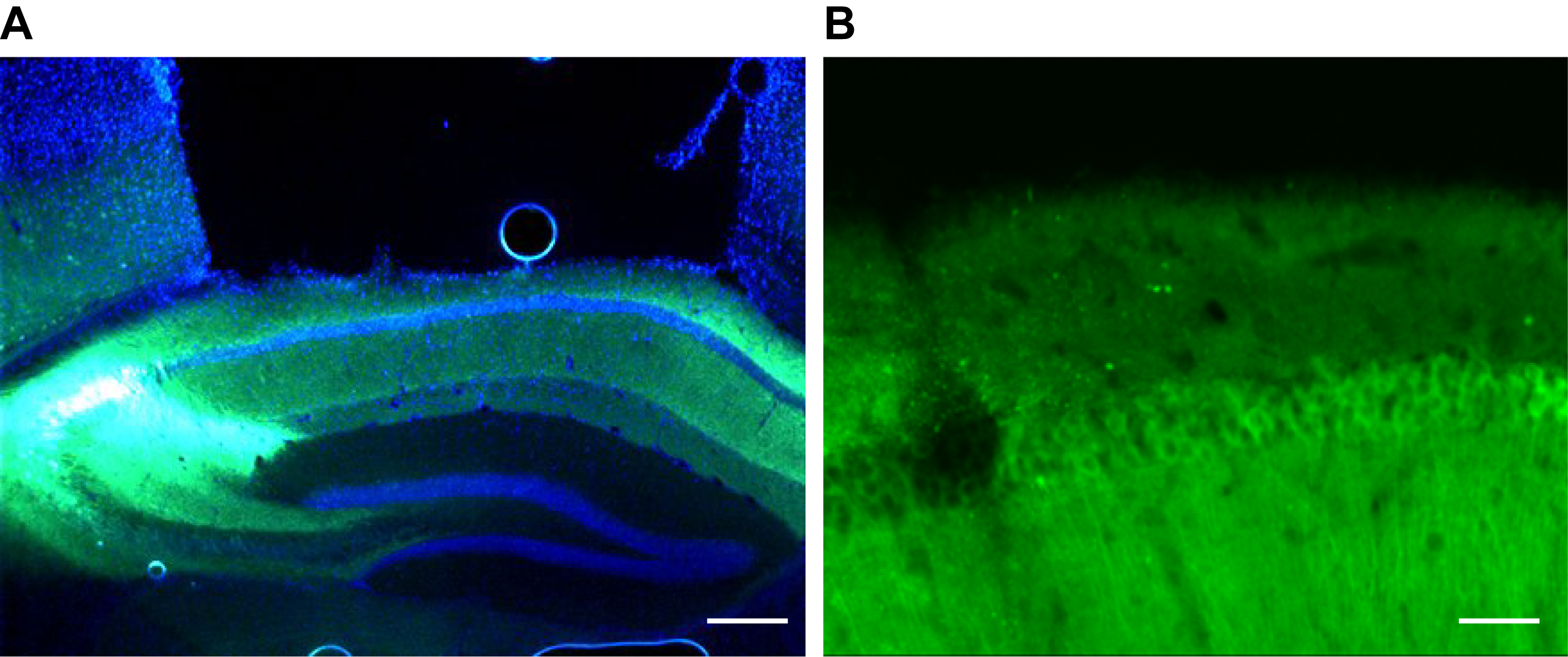

Supplement: Figure S1 — Histological examination of the tissue following the imaging procedures. A: A representative example of placement of the optical cannula over CA1 region of the hippocampus (coronal slice, scale bar: 180 µm). Green: GCaMP3 fluorescence. Blue: DAPI staining. B: A representative example of the GCaMP3 fluorescence (green) under the optical cannula (scale bar: 88 µm). (TIF) [file pone.0112068.s001.tif]

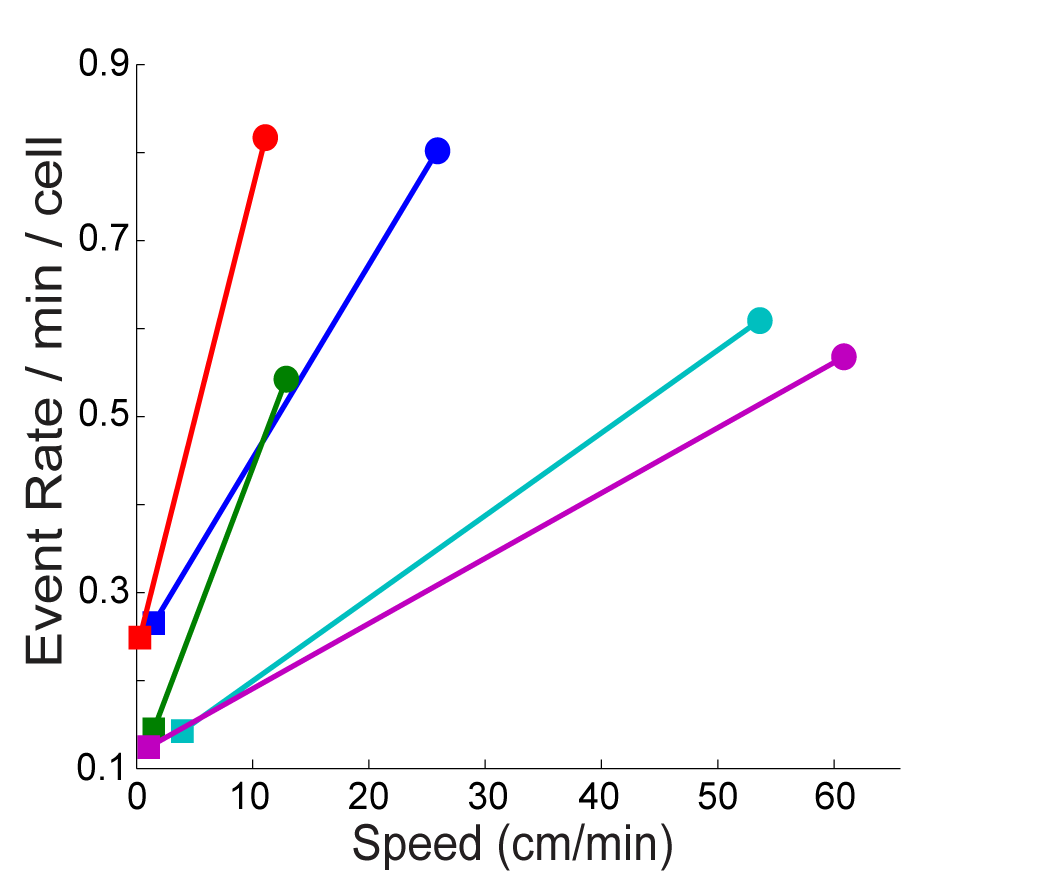

Supplement: Figure S2 — Average rate of calcium transients in all animals used in the study plotted versus average speed of the animals in each condition (indicated by the shape of the marker; vehicle: circles; Zolpidem: squares). (TIF) [file pone.0112068.s002.tif]

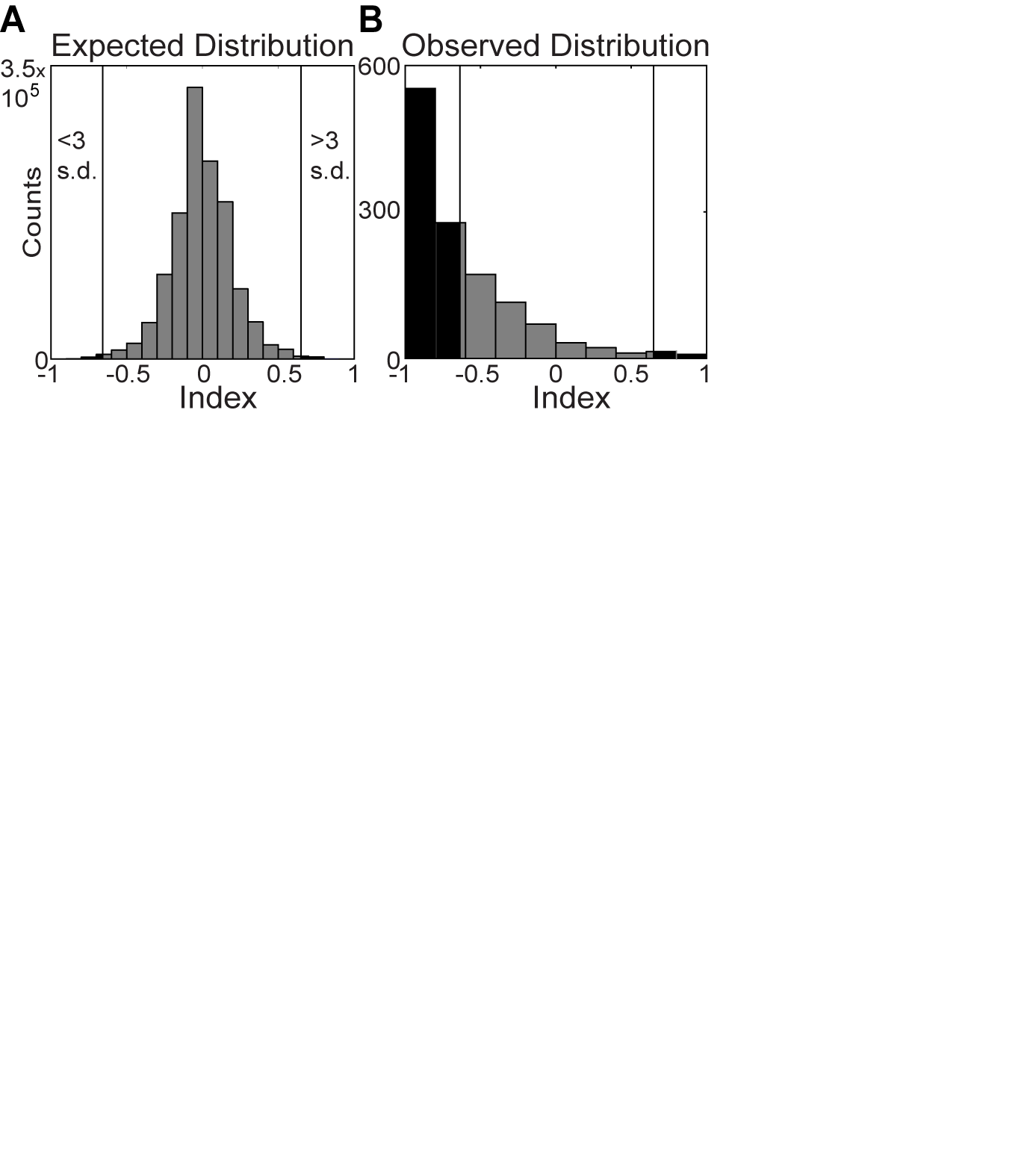

Supplement: Figure S3 — Distributions of normalized drug index: (post-drug event rate - post-vehicle event rate)/(post-drug event rate+post-vehicle event rate). A: The distribution of drug indices expected if Zolpidem had no effect was constructed by re-sampling, with replacement, vehicle data bins within each cells (1000 shuffles). B. The observed distribution of drug indices calculated for each cell (n = 1275). Vertical line indicates 99% confidence interval (3 s.d.) calculated from the expected distribution. (TIF) [file pone.0112068.s003.tif]
